# Supplementary material for: Brain-Derived Neurotrophin and TrkB in Head and Neck Squamous Cell Carcinoma
Source: Int J Mol Sci. 2019 Jan 11;20(2):272. doi: 10.3390/ijms20020272 (PMC6359060; doi:10.3390/ijms20020272)
Supplement: Supplementary file 1 [file ijms-20-00272-s001.zip › ijms-408811-SI/supplementary figure_1.docx]

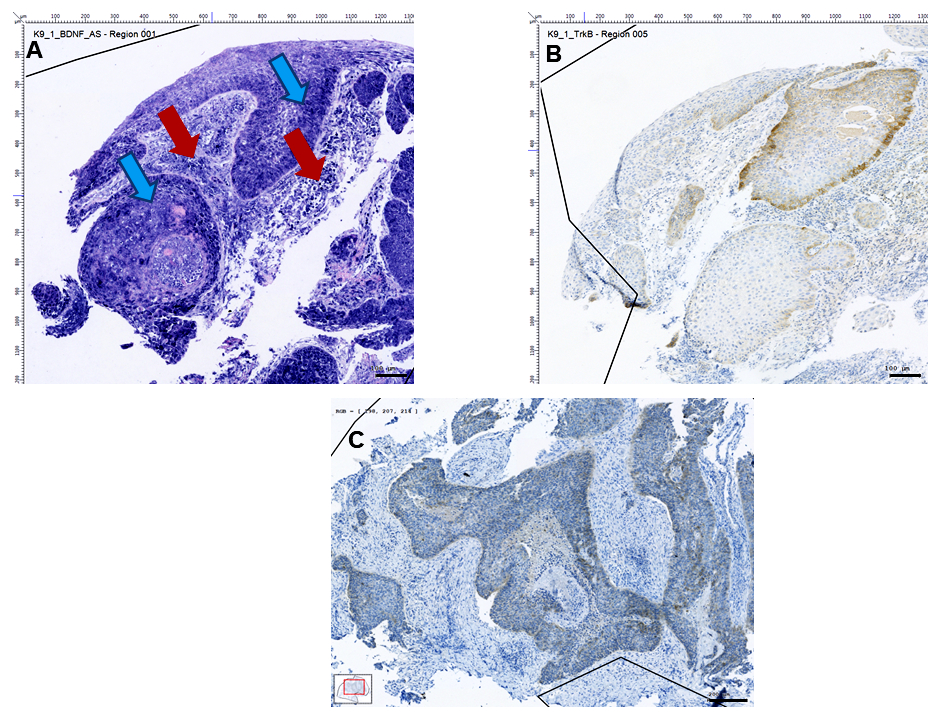


**Supplementary Figure 1. mRNA expression of brain-derived neurotrophic factor (BDNF) in HNSCC (A) and focal (B), or diffuse (C) staining pattern of TrkB protein in HNSCC**

**A)**: In situ hybridisation of antisense riboprobe for BDNF (blue) in larynx SCC, cell nuclei counterstained in nuclear fast red. The antisense probe shows intensive purple – blue reactive areas both in tumor cell nests (blue arrows) and in stroma (red arrows). Two types of staining pattern have been found for TrkB in HNSCC, a focal as displayed on panel **B**, and diffuse as displayed on panel **C**. **A-C** images were taken by the TissueFaxs system, bars: 100 µm: **A-B**; 200 µm: **C**.
