# Supplementary material for: Brain-Derived Neurotrophin and TrkB in Head and Neck Squamous Cell Carcinoma
Source: Int J Mol Sci. 2019 Jan 11;20(2):272. doi: 10.3390/ijms20020272 (PMC6359060; doi:10.3390/ijms20020272)
Supplement: Supplementary file 1 [file ijms-20-00272-s001.zip › ijms-408811-SI/supplementary figure_2.docx]

| Distribution of HPV^+^ and HPV^-^ cases | | Frequency | Percent |
| --- | --- | --- | --- |
| HPV Background | negative (0) | 98 | 74,8 |
|  | positive (1) | 33 | 25,2 |
|  | All | 131 | 100,0 |


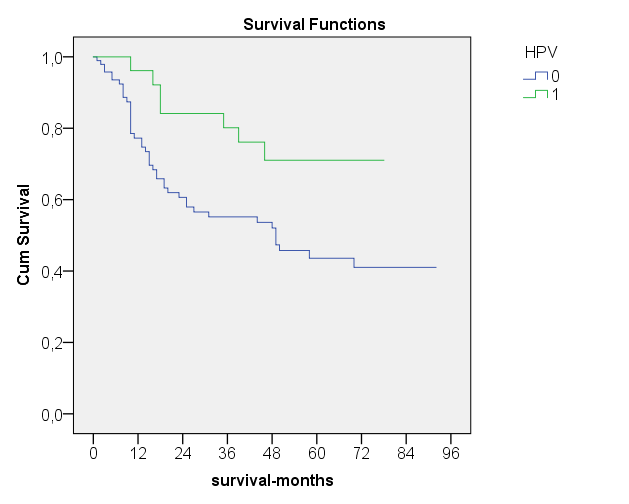


| Means and Medians for Survival Time | | | | | | | | |
| --- | --- | --- | --- | --- | --- | --- | --- | --- |
| HPV | Mean^a^ | | | | Median | | | |
|  | Estimate | Std. Error | 95% Confidence Interval | | Estimate | Std. Error | 95% Confidence Interval | |
|  |  |  | Lower Bound | Upper Bound |  |  | Lower Bound | Upper Bound |
| 0 | 51.295 | 4.224 | 43.016 | 59.574 | 49.000 | 13.521 | 22.499 | 75.501 |
| 1 | 63.184 | 4.887 | 53.605 | 72.763 |  |  |  |  |
| Overall | 56.505 | 3.644 | 49.363 | 63.647 | 70.000 |  |  |  |
| a. Estimation is limited to the largest survival time if it is censored. | | | | | | | | |

| Pairwise Comparisons | | | | | |
| --- | --- | --- | --- | --- | --- |
| HPV | | **0** | | **1** | |
|  |  | Chi-Square | **Sig.** | Chi-Square | **Sig.** |
| Log Rank (Mantel-Cox) | **0** |  |  | 5.872 | **0.015** |
|  | **1** | 5.872 | **0.015** |  |  |
| Breslow (Generalized Wilcoxon) | **0** |  |  | 6.526 | **0.011** |
|  | **1** | 6.526 | **0.011** |  |  |
| Tarone-Ware | **0** |  |  | 6.277 | **0.012** |
|  | **1** | 6.277 | **0.012** |  |  |

| Group | Maximal observation time (months) | Number of cumulative events |
| --- | --- | --- |
| HPV-negative | 92 | 44 of 98 |
| HPV-positive | 78 | 7 of 33 |

**Supplementary Figure 2. Influence of HPV background on patient survival**

Thirty three of 131 patients were HPV^+^, which showed significantly better survival, as displayed in the Kaplan-Mayer survival curves and evidenced by detailed statistical information of Kaplan-Mayer survival analysis and Log-Rank (Mantel-Cox), Breslow and Tarone-Ware pairwise comparisons.
