# Supplementary material for: Brain-Derived Neurotrophin and TrkB in Head and Neck Squamous Cell Carcinoma
Source: Int J Mol Sci. 2019 Jan 11;20(2):272. doi: 10.3390/ijms20020272 (PMC6359060; doi:10.3390/ijms20020272)
Supplement: Supplementary file 1 [file ijms-20-00272-s001.zip › ijms-408811-SI/supplementary figure_3.docx]

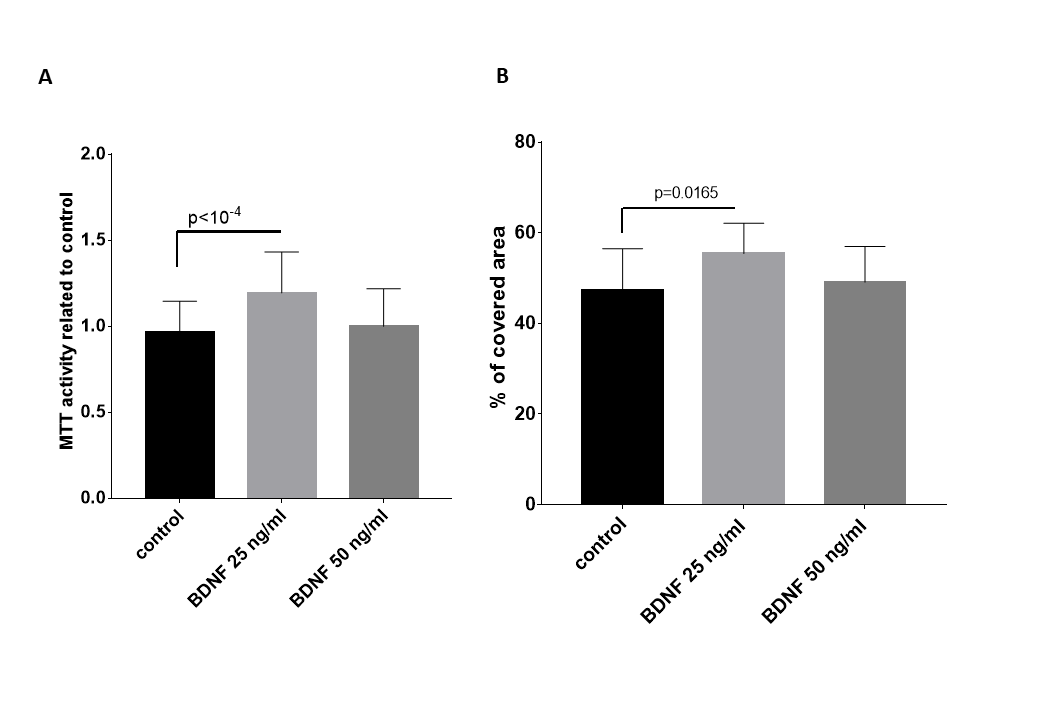


**Supplementary Figure 3. Effects of BDNF on cell growth**

UPCI SCC090 cells were treated with 0-50 ng/ml BDNF for two times 48 hours and cell growth was analysed by MTT assay (**A**) and by analysing the covered area (**B**) using the Juli live imaging instrument. Both methods revealed low, but significant effects by 25 ng/ml concentration, which did not increase by using higher concentration. The column bars represent mean +/- S. E. M. In MTT-assay: N=48 in all categories, not all data were normal distributed using D'Agostino & Pearson normality test. Using Tukey's multiple comparisons test 25 ng/ml BDNF treatment showed significant increase compared to the control levels. In covered area analysis: N=24 in all categories, all data were normal distributed using D'Agostino & Pearson normality test. Using Dunnett's multiple comparisons test

25 ng/ml BDNF treatment showed significant increase compared to the control levels.
